# Insights and recommendations for working collaboratively and improving care in Alzheimer's disease: Learnings from the Finding Alzheimer's Solutions Together (F.A.S.T.) Council

**DOI:** 10.1111/hex.14040

**Published:** 2024-04-17

**Authors:** Jannice Roeser, Nikki Bayliss, Marco Blom, Ruth Croney, Lydia Lanman, Jerson Laks, Marco Lyons, Lea Proulx, Marianna Tsatali, Karin Westerlund, Jean Georges

**Affiliations:** ^1^ F. Hoffmann‐La Roche Ltd Basel Switzerland; ^2^ Alzheimer's Disease International London UK; ^3^ Alzheimer Netherlands Amersfoort The Netherlands; ^4^ Roche Products Ltd Welwyn Garden City UK; ^5^ Federação Brasileira das Associações de Alzheimer Rio de Janeiro Brazil; ^6^ Roche Innovation Center F. Hoffmann‐La Roche Ltd Basel Switzerland; ^7^ Alzheimer Hellas Thessaloniki Greece; ^8^ Stiftelsen Alois Alzheimer Stockholm Sweden; ^9^ Alzheimer Europe Luxembourg Luxembourg

**Keywords:** Alzheimer's disease, dementia, patient‐centred care, patient education, patient engagement, improving dementia care

## Abstract

**Background:**

Collaborations between patient organisations (POs) and the pharmaceutical industry can help identify and address the unmet needs of people living with a disease. In Alzheimer's disease (AD), the scale and complexity of the current unmet needs call for a broad and cross‐sectoral collaboration, including people living with Alzheimer's (PLWA), their care partners and the wider research community.

**Objective:**

This study aimed to describe learnings from the Finding Alzheimer's Solutions Together (F.A.S.T.) Council, a collaboration between POs and Roche, convened to better understand the unmet needs of PLWA and their care partners.

**Results:**

1. Learnings from the collaboration, including clarifying objectives and members' expectations upfront, and establishing a set of guiding values and engagement principles. 2. Insights and recommendations for improving care in AD, including a wide range of unmet needs and potential solutions, systematically captured throughout the PLWA journey. These have resulted in several published reports and other outcomes, including (1) ‘Portraits of care’, highlighting the role of care partners, and the impact of coronavirus disease 2019 on care; (2) Clinical trial guidebook, recommending how PLWA and care partner experience can be incorporated into trial design; (3) ‘Commitments Catalogue’, highlighting progress by governmental organisations in achieving their commitments; and (4) a report to guide policy on improving diversity, equity and inclusion in clinical trials.

**Conclusions:**

Close collaboration between POs and the pharmaceutical industry in AD can enable effective research, in which PLWA and care partners are engaged as ‘experts through experience’ to help identify key unmet needs and co‐create solutions with the wider AD research community. This paper and the work undertaken by the F.A.S.T. Council may act as a blueprint for meaningful collaboration between POs and the pharmaceutical industry.

**Patient or Public Contribution:**

The paper reports the collaboration between POs, the F.A.S.T. Council and Roche to progress towards a future in which PLWA can live fulfilling lives with their disease managed well.

**Clinical Trial Registration:**

Not applicable.

## 1 INTRODUCTION

Alzheimer's disease (AD) is a progressive neurodegenerative disease that damages healthy cells in the brain, causing cognitive impairment and functional disability.^1^ AD is the most common cause of dementia and a growing global health concern with huge implications for individuals and society.^2^ It is estimated that around 40 million people worldwide suffer from AD.^3^ This is predicted to more than triple by 2050 as the population ages.^4^ The societal and economic cost of dementia in Europe is estimated to increase to over €250 billion by 2030.^5^

Common symptoms include memory and cognitive deficits, and personality and behaviour changes.^6^, ^7^ As the disease progresses, AD significantly impacts the lives of people living with Alzheimer's (PLWA) and can have devastating effects on their care partners and loved ones.

The AD community faces significant unmet needs.^8^ Although scientists have made tremendous progress in better understanding AD in recent years, there are limited effective treatment options available that modify, halt or slow the course of disease.^9^ Recently, we have seen the first traditionally approved treatment that addresses the underlying biology of AD and changes the course of the disease in a meaningful way for people in the early stages.^10^ However, significant unmet needs remain, for example, identifying people with early AD, and treating more advanced stages of disease.^11^

There are also significant gaps in the AD care pathway, such as lack of funding for specialist testing; shortage of clinical assessment tools that measure the effectiveness of therapies in early AD; absence of centralised knowledge and support; and lack of appropriate clinical trial infrastructure and scarcity in patient registries to support rapid enrolment in clinical trials.^12^, ^13^

In addition, continued stigma and lack of awareness amongst the general population and general practitioners (GPs) constitute further barriers to timely detection.^14^

The scale and complexity of AD call for broad, cross‐sectoral collaboration that mobilises the skills, knowledge and experiences of the AD community. In particular, there is a need to better understand the disease impact from the perspectives of PLWA and their care partners to develop a full understanding of AD over time to help shape the environment to better meet the needs of those impacted by the disease.^15^

PLWA, care partners and patient organisations (POs) are key stakeholders in this process as they can provide the ‘lived experience’ of AD, helping provide a true picture of what it is like to live with AD; how care is delivered and how it impacts PLWA, their care partners and their families; and how treatments may impact their quality of life. PLWA and POs are ‘experts by experience’ and are now more equipped than ever to define healthcare in their own terms.

For the pharmaceutical industry, partnering with patients and POs to further understand their everyday lives is essential to discover insights and develop solutions that fulfil the unmet needs of people living with a disease.

Close collaboration with POs can take the form of ‘public involvement in research’, which engages those within the community of interest in the research process itself. The results can then be prioritised and applied to the wider community.

In 2020, Roche convened the Finding Alzheimer's Solutions Together (F.A.S.T.) Council, a bi‐annual meeting comprising several AD POs and Roche employees. The primary objective of the F.A.S.T. Council was to better understand the perspectives and unmet needs of PLWA and their care partners. This paper outlines the insights gathered by the F.A.S.T. Council between 2020 and 2023, as well as recommendations on how POs and the pharmaceutical industry can successfully collaborate to co‐create and deliver patient‐focused solutions. The members of the F.A.S.T. Council hope that these learnings can be leveraged by other organisations within the AD community.

## 2 METHODS

Seeking to expand its understanding around the unmet needs of PLWA to identify potential solutions where Roche could add value, Roche established and funded the F.A.S.T. Council and invited several AD POs to become members. The membership evolved over time depending on requirements and time resources. A core group of members consistently remained on the F.A.S.T. Council, listed in alphabetical order:

- Alzheimer's Disease International, UK.
- Alzheimer Europe, Luxembourg.
- Alzheimer Hellas, Greece.
- Alzheimer Nederland, the Netherlands.
- CanAge: Canada's National Seniors Advocacy Organization, Canada.
- Federação Brasileira das Associações de Alzheimer (Febraz), Brazil.
- Shanghai Jian'ai Charity Organization, China.
- Stiftelsen Alois Alzheimer, Sweden.

Organisations representing other countries—including South Africa and the United States—were represented on an ad hoc basis.

The agreed primary objective of the F.A.S.T. Council was to better understand the perspectives and unmet needs of PLWA and their care partners, with a particular focus on early detection, diagnosis, clinical trials information, access to new therapies, policy and political advocacy and health system preparedness. Roche aimed to identify areas where it could provide further support to help improve the overall AD diagnostic and treatment journey for PLWA and improve the experience of PLWA and care partners participating in clinical trials. The F.A.S.T. Council acknowledged that there would be differences and similarities across countries in AD care. The F.A.S.T. Council agreed upon a series of core values—trust, transparency, integrity and independence—forming the foundation of the collaboration and defining how the F.A.S.T. Council members would operate, behave and interact together.

In the early stages of the collaboration, the F.A.S.T. Council was set up with the support of an independent third‐party agency (Ipsos).

Clear and transparent contracting, including confidentiality rules and agreements, was agreed to and signed by all parties. All POs received honoraria to reimburse their time and in recognition of the value that their participation provided.

These ground rules, as well as the moderation of meetings by Ipsos, ensured full transparency, equality of involvement and trust between the PO members and Roche.

The F.A.S.T. Council convened a total of six times between 2020 and 2023. Meetings were maximum 3 h in duration, with regular opportunities for questions or breaks.

As a global collaboration, meetings were conducted virtually. To optimise communications, members used their video function and online digital features (e.g., chat, Q&A, breakout rooms, reaction icons), which can promote feedback, inclusiveness and co‐creation.

To maximise the time, slides were shared before meetings, along with surveys to identify positions on selected topics. As well as open group discussions, breakout groups were formed to facilitate detailed discussion on specific key questions, such as, ‘Where do you see the greatest unmet needs in the field of AD?’ and, ‘What is your vision for the future of AD care?’ Breakout participants were encouraged to feed back to the larger group to gain consensus on insights.

Minutes were sent to members after each meeting. Over time, the insights from the meetings were distilled into the AD patient journey in a systematic way, with alignment on the key unmet needs, optimum patient journey and potential strategies or actions at each stage of the journey. The outcomes of this process are outlined in this paper.

Following the news in November 2022 that gantenerumab, Roche's investigational molecule for the treatment of AD, did not reach its primary endpoint in phase 3 clinical trials,^16^ the F.A.S.T. Council convened to discuss the future role of the Council.

Roche remains committed to working with the patient community in AD and the F.A.S.T. Council, but suggested that the focus of collaboration with PO should shift to early research and development initiatives. The F.A.S.T. Council unanimously agreed that the learnings, recommendations and outputs from its meetings to date should be shared with the wider AD community.

## 3 RESULTS

The results and learnings from the F.A.S.T. Council meetings are divided into two main areas: (1) learnings from the collaboration itself, resulting in best practice recommendations for industry–PO partnerships; and (2) insights and recommendations for improving care in AD.

### 3.1 Industry–PO collaboration: Best practice recommendations

The F.A.S.T. Council used a systematic, transparent and inclusive approach to its work, which fostered a highly collaborative working environment. Three ways of working are recommended for other industry–PO partnerships.

#### 3.1.1 Clarify objectives and members' expectations

Inevitably, some divergent and overlapping interests and priorities will exist between pharmaceutical companies and POs, making it vital to identify shared goals and expectations.

Council members were asked to identify their priority areas and expectations for the F.A.S.T. Council. Feedback from all members was collected. Areas of clear alignment with the greatest potential impact for PLWA, the AD community and Roche were prioritised.

Once overall objectives were agreed, the roles and responsibilities of Council members were decided, with each member committing to contribute specific areas of focus.

#### 3.1.2 Establish a set of guiding values

The F.A.S.T. Council defined and agreed to a set of values that would be used to guide the collaboration: trust, transparency, integrity and independence. The perspectives of all members were respected, recognising the diversity of expertise and insights of POs. Roche took primary responsibility to ensure that legal and ethical guidelines were followed. It was equally important for the PO community to understand their responsibilities, particularly in terms of safeguarding confidentiality with sharing of information.

These values served as a reference point to inform discussions and address any potential misalignment. The resulting working culture was open, inclusive, personable, interactive and objective‐driven.

Roche was keenly aware that some Council members were balancing multiple responsibilities—for example, the professional responsibilities involved in running a PO—meaning that the F.A.S.T. Council needed to be empathetic and flexible to accommodate the time and needs of participants.

#### 3.1.3 Establish engagement principles for virtual working

Most meetings took place during coronavirus disease 2019 (COVID‐19), so the following tools were utilised to enable meaningful virtual collaboration.

1. *Shared decision‐making*, for example, the members co‐created the F.A.S.T. Council's name, and formalised the common goals and values.
2. *Digital engagement*, for example, the use of online bulletin boards, digital forms, polls and video sharing.
3. *Individual reflection*, for example, active and passive listening was encouraged, and space was provided for quiet reflective thinking as the topics discussed could be emotionally difficult.
4. *Group breakout sessions*, for example, members were encouraged to provide insights in small peer groups.
5. *Accessible materials*, for example, the outputs from meetings were captured as illustrations, to ensure that these would be engaging and easily accessible by the AD community.

### 3.2 Insights and recommendations for improving care in AD

The insights and recommendations for improving care in AD have been broadly divided into four separate topics:

- Optimising the PLWA and care partner journey.
- Giving PLWA and care partners a voice.
- Enabling policy and healthcare system readiness.
- Integrating the perspectives of PLWA into clinical trial design.

#### 3.2.1 Optimising the PLWA and care partner journey

One of the main areas of focus for the F.A.S.T. Council was to better understand the current challenges experienced by PLWA, their families and care partners throughout the patient journey. To achieve this, members engaged directly in the Council's work in their role as ‘experts through experience’, and identified unmet needs and potential solutions.

Over the course of the meetings, unmet needs, desired future states and potential solutions were captured via the meeting minutes. It was agreed between the group that these insights should be captured in a systematic way, which could be shared externally. The Council agreed to illustrate the key issues affecting PLWA and their care partners through the four identified stages of the patient journey: prediagnosis, diagnosis, treatment and disease management.

Each stage of the patient journey was populated with key unmet needs, the ‘desired state’, identified strategies or actions that may help achieve this desired state and then either existing solutions where known, or new potential solutions as proposed by members of the F.A.S.T. Council. As insights and solutions were collated, F.A.S.T. Council members had the opportunity to review, edit and supplement them further.

The results of this process can be found in Table 1.

**Table 1:** Unmet needs and potential solutions throughout the four key stages of the PLWA journey.

Stage of journey	Unmet need	Desired state	Identified actions	Existing/proposed solutions
Prediagnosis	High stigma/lack of awareness. Lack of access to diagnosis opportunities for those living in remote areas	Stigma reduced, brain health openly discussed	Educate the general public	Existing: ADI and Pan American Health Organization created the ‘Let's talk about Dementia’ campaign, available in five languages^17^
			Enable self‐assessment	Existing: SAGE^18^
			Provide reassurance for at‐risk populations	New: Use digital channels to share positive messages, personal stories
		Digital and other tools are available to help people look after their brain health and be aware of AD symptoms	Make it easier to find support solutions when PLWA have neurological concerns	New: Use digital tools to provide better support, enhance lifestyle and preventative behaviour
			Educate GPs to recognise signs early	Existing: Telemedicine and virtual platforms, for example, Project ECHO,^19^ can help GPs New: Share info for clinicians in engaging ways (e.g., gamification)
Diagnosis	Initial assessment: Some clinicians ‘watch and wait’, leading to delays	Clinicians sensitively explore AD diagnosis, providing counsel and support	Ensure the right people go to the GP/specialist at the right time for the individual	Existing: Alzheimer's Society online symptoms checker^20^ New: Provide validated self‐assessment tools to trigger GP visit, for example, self‐administered screening tool to detect mild cognitive impairment New: Access to digital biomarkers to detect biological changes before cognitive impairment
	Referral: Lengthy delays until symptoms worsen	Effective and clear pathways in a timely manner		
	Diagnosis: Sometimes delays. Some care partners may feel guilt for not acting sooner	Confirmed via PET scan or cerebrospinal fluid test	Facilitate specific and accurate diagnosis	
	What next: Lack of information for families on journey ahead	Clinicians are trained to discuss pathway and what to expect	Ensure ongoing engagement with specialists and GPs post‐diagnosis	New: Develop mobile app to remotely track changes to ensure timely contact with HCPs
			Promote access to trusted resources	
Treatment	Are PLWA receiving the right treatment (or treatment + lifestyle/psychological interventions) at the right time?	Disease‐modifying therapies available to all PLWA who will benefit from them	Ensure that information is provided on treatment availability and applicability	New: A virtual reality tool for care partners and PLWA to prepare for the experience of treatments New: An integrated information, education and communication platform that uses gamification to increase interaction and engagement
			Ensure that information is provided on how to monitor ongoing impact of treatment	
			Enable treatment scheduling and consistent follow‐up	
			Enable consistent availability of treatments across countries	
			Ensure that GPs are aware of latest developments	
		Clinicians include PLWA on standardised registries to collect real‐world evidence		
		Expectations of PLWA are well managed. PLWA are given clinical trial opportunities		
Disease management	Treatment continuation: Care partners often suffer financial hardship and do not have access to community support	PLWA and care partners are informed and understand the benefits of treatment. Monitoring is in place	Ensure consistent availability of disease management tools, especially for late‐stage AD	Existing: Disease management and monitoring digital tools, such as ALZNavigator^21^—an interactive online tool that creates custom action plans for individuals and families based on their current situation Existing: Remote monitoring and AI tools such as the ICA^22^—a remote test based on humans' strong reaction to animal stimuli
			Enable PLWA to maintain independence, with support for those living alone	Existing: Remote caregiving tools, such as appliances that turn off lights and JustChecking^23^
			Provide wrap‐around psychosocial care	
		Clinicians openly discuss disease progression. Care partners receive community support	Enable PLWA and care partners to communicate with others in a similar position	Existing: AlzConnected, a platform for PLWA and care partners to form social connections^24^
			Ensure that care partners receive support to help them look after PLWA	New: Ensure that digital companion tools are tailored to individual situations and provide meaningful solutions based on input from other care partners
	Palliative care: PLWA at home increases financial and emotional impact on care partners. Care institutions/care homes are not always set up to deal with AD	Support for PLWA of all socioeconomic statuses is available. Institutions adapt to care for those with severe AD	Ensure that HCPs understand the impact of comorbidities	New: Use AI to connect activity monitoring tools to broader technologies and clinicians to provide HCPs with the big picture to understand which element of any current lived experience requires the most urgent attention

Abbreviations: AD, Alzheimer's disease; ADI, Alzheimer's Disease International; AI, artificial intelligence; GP, general practitioner; HCP, healthcare professional; ICA, Integrated Cognitive Assessment; PLWA, people living with Alzheimer's; SAGE, Self‐Administered Gerocognitive Examination.

#### 3.2.2 Giving PLWA and care partners a voice

Living with AD can be understood as becoming entangled in uncertainty and isolation.^25^ PLWA and their care partners often feel sidelined from decision‐making about their condition. People living with cognitive impairment often have the desire and ability to participate in decision‐making about their everyday care, although this is regularly underestimated by their professional colleagues and care partners.^26^ POs are in need of accurate and detailed updates on research from the pharmaceutical industry. Very often, they are the ones needed to interpret complex messages to PLWA and their care partners.^27^

##### Developing a standardised lexicon

It is important that PLWA and their care partners have a voice in their own care and that others use empowering and respectful language when talking about, and to, PLWA. It was agreed that having a standardised lexicon for AD in place would support this aim.

A number of F.A.S.T. Council members hosted a series of workshops with Roche's CareRing network (a global internal community for employees who either live with a disease or have experience providing care for a particular disease) to discuss appropriate language to use with PLWA. Five key recommendations were made for the development of an AD‐appropriate lexicon:

1. *Individual focused*: Use inclusive language to respect identity and sense of self.
2. *Empowerment and personhood*: Use language that respects people's preferences and highlights their strengths and abilities.
3. *Communication*: Use language that supports changes or overcomes barriers in communication.
4. *Effect of AD*: Use language to support changes in personal experience.
5. *Emotional journey*: Use language that supports resilience and frames the positives.

Language, social and cultural differences all affect the wording used and meaning perceived around a particular topic.^28^ As such, the F.A.S.T. Council recommended a lexicon that is flexible and contextually appropriate.

##### Research exploring support and coping strategies of care partners of people living with AD

Caring for someone who is living with AD can be a rewarding role; however, it can also come with significant challenges, such as high stress, loneliness and loss of identity.^29^, ^30^ In comparison with care partners of people with other long‐term conditions, care partners of PLWA often have poorer physical and emotional health.^31^

The COVID‐19 pandemic exacerbated the situation, further impacting the mental and physical health of PLWA and their care partners.^32^

Roche commissioned a survey—‘Portraits of care’—which was undertaken in partnership with ADI, a F.A.S.T. Council member. Care partners from several countries were interviewed about their experience of caring for someone living with AD. This survey explored the impact of COVID‐19 restrictions on AD care, ultimately seeking to improve structural and social support for care partners and PLWA.

Participants were asked to bring photographs and/or other visual images to the interview, to represent their perceptions or experiences. The use of visual images leads to richer and deeper accounts and more emotional and meaningful responses.^33^, ^34^

The research identified three typologies that reflect the ways in which different people approach their care partner role:

- *Empaths* (Feeling): Compassion, empathy, loving acts of care, pride, growth, routine and learning. They primarily have emotional challenges.
- *Organisers* (Thinking): Perspective, objective descriptions of tasks, routine, expertise and pride. Their challenges are often organisational.
- *Nonidentifiers* (Reluctance): No life, no support, difficulty adapting, trapped and alone. Face major challenges with accepting their role as a care partner.

The typologies, agreed with by the F.A.S.T. Council, can help care partners self‐identify and give them a voice, helping them express their experiences of caring for someone with AD, for example, via digital interaction tools, for example, AlzConnected, as identified in the optimal care partner journey. A poster outlining the 'Portraits of care' initiative was presented at the Alzheimer Europe Conference 2022.^35^

#### 3.2.3 Enabling policy and healthcare system readiness

There is an urgent need for AD to be prioritised by governments and to prepare health systems to provide access to early detection, timely diagnosis and care.^36^

Members of the F.A.S.T. Council identified two key priority policy areas:

1. Getting dementia back on global, regional and local political and policy agendas.
2. Driving the adoption and implementation of comprehensive national dementia plans.

##### ‘Commitments Catalogue’ research

The F.A.S.T. Council appointed a focused Policy Squad, tasked with cataloguing commitments to AD made by supranational and governmental organisations and determining to what extent these commitments have been upheld.

Insights from the ‘Commitments Catalogue’ research showed that while AD is present on global agendas, meaningful progress has been slow, with national governments largely off track in achieving their stated policy goals, including those stated in the World Health Organization's (WHO) Global Action Plan on Dementia.^37^ Unlocking dedicated funding for AD is a challenge as health systems face strains. While national strategies and action plans are in place in some countries, progress updates are infrequent and existing strategies often lack dedicated funding and indicators for progress.^38^

##### Tracking progress of government action on AD

Council members shared some of their work in benchmarking and tracking governmental progress in AD and issuing calls for action to fulfil unmet needs. In its World Alzheimer's Report 2022, ADI calls for National Dementia Plans to become a policy priority.^39^ ADI produces an annual From Plan to Impact report to track progress against the WHO's Global Action Plan. The fifth edition in 2022 noted that only 39 WHO member states have developed national dementia plans, despite all 194 member states committing to developing such policies in 2017.^40^ ADI has since called on the WHO to prolong the deadline set out in the Global Action Plan by 4 years, to 2029.

In its 2022 Dementia Report, CanAge reviews Canada's National Dementia Strategy and calls for investment to increase the numbers of geriatricians per person above 65 years of age and for Canadian provinces to develop clear dementia strategies, action plans or care pathways for those living with dementia.^38^ Meanwhile, Alzheimer Europe's Dementia Monitor compares and benchmarks national dementia policies and strategies through assessment and ranking of readiness across 10 areas, including care and support services.^41^

#### 3.2.4 Integrating the perspectives of PLWA into clinical trial design

Recruitment for, and participation in, AD clinical trials has historically been a challenge.^42^, ^43^ Several barriers to trial participation have been reported.^44^, ^45^

At every disease stage, AD trials require the enrolment of both a participant and a study partner—the study partner is a person who commits to the clinical trial and acts as the knowledgeable informant. Most of the time (but not always), the study partner is the existing care partner (usually a spouse or child).^46^

Insights were provided by PLWA and their study partners before, during and post clinical trial participation; healthcare professionals involved in clinical trial management; and global and local POs, including the European Working Group of People with Dementia, who provided invaluable input on research protocols, the organisation and frequency of study visits and materials for study participants and study partners.

The barriers reported include the following:

- Lack of a study partner to support the PLWA throughout the trial and to note cognitive changes or treatment effectiveness.
- Pain and fear associated with undergoing invasive procedures during a trial.
- Distance to the trial centre and travel costs.
- Time commitment necessary from both the participant and their study partner.
- Concern over the risks to which participants may be exposed.
- Participants' comorbidities.
- Lack of history of positive effects in AD clinical trials.
- Lack of ability to engage with underrepresented populations.
- Lack of knowledge about the implementation of clinical trials in the medical community.
- Lack of representation of PLWA with comorbid disabilities (e.g., people who are deaf or hard of hearing are often excluded in clinical trials).

##### Clinical trial guidebook: Integrating the perspectives of PLWA

Recognising that AD clinical trials can only be successful if they fully embrace meeting the needs of PLWA and their care partners, the F.A.S.T. Council developed a comprehensive guidebook supporting clinical trial design and management to encompass the needs of participants and their study partners.^45^

In the guidebook, the F.A.S.T. Council asserts that, given the challenges and barriers facing participant recruitment into, and retention in, AD clinical trials, there is a need to rethink the design and delivery of clinical trials to ensure that they consider the needs of PLWA and their care partners throughout. The guidebook includes a range of recommendations—from the importance of enabling connection on a personal level with PLWA and study partners to build trust, to practical support during a trial, such as using remote assessments where possible, as well as covering any costs associated with travel, and helping justify absences from work, for example, through a letter of support. Roche presented on the Clinical trial guidebook initiative—and the contributions of the F.A.S.T. Council—at the Alzheimer Europe Conference 2022. The full recommendations can be found in the report online.^45^

##### Diversity, equity and inclusion

In AD clinical trials, there is inadequate engagement of people from minority racial and ethnic backgrounds, who have historically been underrepresented in clinical research.^47^

During F.A.S.T. Council meetings and discussions, the importance of diversity, equity and inclusion (DE&I) in AD clinical trials was repeatedly identified. The burden of dementia falls disproportionately on certain groups; for example, AD has a disproportionate impact on women compared with men,^48^ while dementia incidence rates are higher for African American and Hispanic populations compared with White populations.^49^

A dedicated report on DE&I—based on research and input from F.A.S.T. Council members and academic and clinical experts—has been developed to help guide policy improvements to ensure DE&I in AD clinical trials.^50^

## 4 DISCUSSION

AD is one of the biggest public health challenges of our time, impacting millions of people and their families around the world.^2^ The care and support for PLWA and their care partners are extremely complex, and their provision is challenging.^51^

There is an urgent need for AD to be prioritised by all stakeholders, to improve the care and support given to PLWA and their care partners.

In addition, the entry of disease‐modifying therapies will require a transformation of the PLWA and care partner pathway, which in turn will require concerted political commitment, so that those who are eligible for treatment are able to access it once these therapies are approved and available.

In this context, collaboration in AD is more important than ever. The F.A.S.T. Council embodied elements of best practice collaboration between POs and the pharmaceutical industry, which may offer a template for future partnerships.

Meanwhile, the insights and recommendations for improving care in AD that resulted from the F.A.S.T. Council offer a combination of ready‐to‐use research and tools (such as the Clinical trial guidebook and the 'Portraits of care' research) and insights and recommendations that may provide a foundation for future activity (such as the Patient journey and AD lexicon work).

Members of the F.A.S.T Council are keen to continue the collaboration and scientific knowledge creation process, on topics including the future needs of people with young‐onset dementia in the development of DMTs for cognitive decline, and the evolution of public involvement in research post‐COVID‐19. The Council will continue its meetings and work collaboratively to collect data and observations, with learnings presented and shared with the AD community.

It is hoped that the learnings from the F.A.S.T. Council will be leveraged and taken forward by other organisations from across the AD community and beyond. Only through collaboration between the pharmaceutical industry, POs and the whole AD community will we create the best opportunities to improve the lives of PLWA and their care partners, and to discover and operationalise the treatments and diagnostics needed to manage this devastating condition.

## 5 LIMITATIONS

The F.A.S.T. Council included representatives from eight different AD POs and one pharmaceutical company, with geographic representation from nine countries. As such, it should be noted that these recommendations may not always be fully reflective of the needs of all PLWA or their care partners. AD is a unique journey that is dependent on many factors, including the local healthcare ecosystem, the stage of the disease and the circumstances of the PLWA and their care partners. The impact of the COVID‐19 pandemic should also be noted, as the time needed to adapt and evolve the F.A.S.T. Council's ways of working during this period may have impacted the knowledge creation process.

## 6 CONCLUSION

Close collaboration between POs and the pharmaceutical industry in AD can enable effective research, in which PLWA and care partners are engaged as ‘experts through experience’ to help identify key unmet needs and co‐create solutions with the wider AD research community.

This paper and the work undertaken by the F.A.S.T. Council may act as a blueprint for meaningful collaboration between POs and the pharmaceutical industry.

## AUTHOR CONTRIBUTIONS

**Jannice Roeser**: Conceptualisation; methodology; investigation; supervision; project administration; writing—review and editing. **Nikki Bayliss**: Writing—review and editing. **Marco Blom**: Writing—review and editing. **Ruth Croney**: Writing—review and editing. **Lydia Lanman**: Writing—original draft; Writing—review and editing. **Jerson Laks**: Writing—review and editing. **Marco Lyons**: Writing—review and editing. **Lea Proulx**: Conceptualisation; methodology; writing—review and editing. **Marianna Tsatali**: Writing—review and editing. **Karin Westerlund**: Writing—review and editing. **Jean Georges**: Writing—review and editing.

## CONFLICT OF INTEREST STATEMENT

Jannice Roeser and Lea Proulx are employees of, and may own stock/stock options in, F. Hoffmann‐La Roche Ltd. Ruth Croney and Marco Lyons are employees of Roche Products Ltd. and may own stock/stock options in F. Hoffmann‐La Roche Ltd. The remaining authors declare no conflict of interest.

## Data Availability

Data sharing is not applicable to this article as no data sets were generated or analysed during the current study.
